# Giant mitochondria in cardiomyocytes: cellular architecture in health and disease

**DOI:** 10.1007/s00395-023-01011-3

**Published:** 2023-09-29

**Authors:** Amy Li, Gerald J. Shami, Lisa Griffiths, Sean Lal, Helen Irving, Filip Braet

**Affiliations:** 1 Department of Rural Clinical Sciences, La Trobe Rural Health School, La Trobe University, Bendigo, VIC Australia; 2 Centre for Healthy Futures, Torrens University Australia, Surry Hills, NSW Australia; 3 School of Medical Sciences (Molecular and Cellular Biomedicine), The University of Sydney, Camperdown, NSW Australia; 4 Australian Centre for Microscopy and Microanalysis, The University of Sydney, Camperdown, NSW Australia; 5 Anatomical Pathology, PathWest, QEII Medical Centre, Nedlands, WA Australia; 6 School of Medical Sciences, The University of Sydney, Camperdown, NSW Australia

**Keywords:** Cardiomyopathy, Giant and megamitochondria, Histopathological marker, Muscle disease, Mitochondrial aberrations, Mitochondrion pathophysiology

## Abstract

Giant mitochondria are frequently observed in different disease models within the brain, kidney, and liver. In cardiac muscle, these enlarged organelles are present across diverse physiological and pathophysiological conditions including in ageing and exercise, and clinically in alcohol-induced heart disease and various cardiomyopathies. This mitochondrial aberration is widely considered an early structural hallmark of disease leading to adverse organ function. In this thematic paper, we discuss the current state-of-knowledge on the presence, structure and functional implications of giant mitochondria in heart muscle. Despite its demonstrated reoccurrence in different heart diseases, the literature on this pathophysiological phenomenon remains relatively sparse since its initial observations in the early 60s. We review historical and contemporary investigations from cultured cardiomyocytes to human tissue samples to address the role of giant mitochondria in cardiac health and disease. Finally, we discuss their significance for the future development of novel mitochondria-targeted therapies to improve cardiac metabolism and functionality.

## Introduction

Mitochondria are dynamic double-membraned intracellular organelles universally recognized as the powerhouse of eukaryotic cells. The heart possesses the highest abundance of mitochondria of any organ tissue where it is estimated to occupy approximately a third of the total cardiac cell volume [53]. Comparatively, cardiomyocytes themselves constitute approximately 70% of the myocardial volume [82]. The primary function of this organelle is to synthesize adenosine triphosphate (ATP) via oxidative phosphorylation. In the myocardium, around 90% of the ATP produced is quickly consumed to maintain heart function and contractility including myosin ATPase to enable actin to detach from the myosin cross-bridge post-power stroke, ATP-dependent sequestration of calcium back into the sarcoplasmic reticulum and in ATP-dependent membrane transport systems. As such, an increase in heart rate and mechanical contractility also increases myocardial oxidative metabolism and energy production by cardiac mitochondria [49]. Alterations in mitochondrial morphology and abnormalities of their internal structures are well documented across the various heart failure phenotypes, including cases of mitochondrial mutations resulting in multi-systemic disease and mitochondrial cardiomyopathy [3, 31, 50, 68]. The heterogeneity in mitochondrial morphology amongst the same disease phenotypes complicates assigning simple and direct structure to function associations.

The human body produces roughly 65 kg of ATP daily that is rapidly utilized as an energy source across all organs. Notably, the heart is responsible for around 8% of total ATP consumption even though it accounts for only 0.5% of total body weight [10]. This gives the heart its status as one of the most metabolically active and energy-demanding organs in the body, preceded only by the liver and brain. Surprisingly, for such a vital organ, it stores only enough energy to support whole heart function over several beats, turning over the entire pool of ATP every 10 s under basal resting conditions [41]. This means that the cardiac mitochondria must continuously generate high concentrations of ATP, and be able to match ATP supply instantly and accurately to metabolic energetic demands of the contractile units under variable physiological conditions. This process of matching energy supply to demand is vital for a range of cellular processes directly attributed to the morphological and functional plasticity of the mitochondria. Furthermore, cardiac mitochondria are not only responsible for ATP biogenesis but also regulate diverse functions in the heart including metabolism, ion homeostasis, and apoptotic processes. These functions are met by the mitochondria’s adaptive capability to alter its size and shape in response to environmental stimuli [75].

**Fig. 1:**
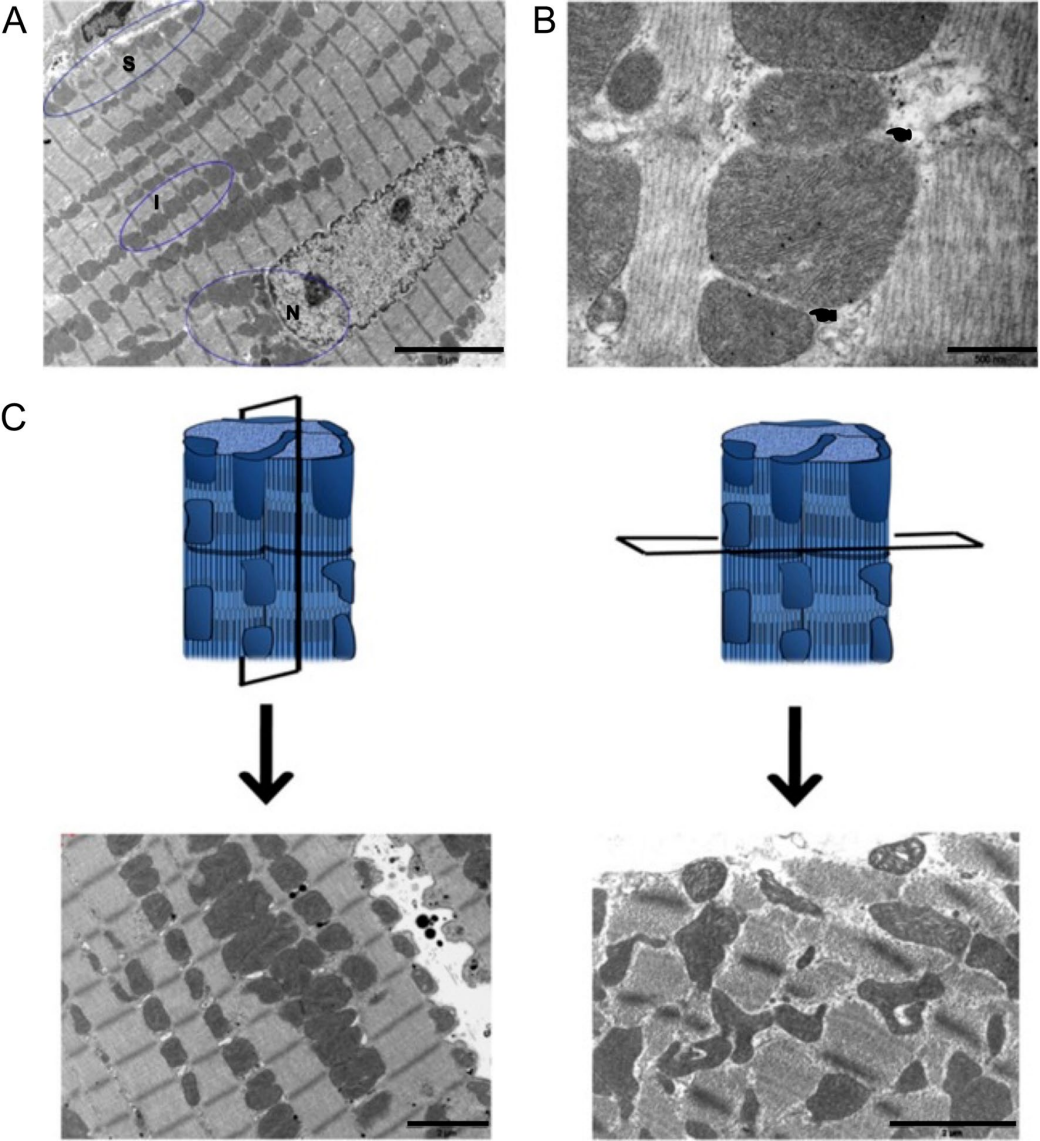
Morphology of mitochondria in the adult ventricular myocardium based on subcellular organization. **A** shows highly organized intermyofibrillar (I) mitochondria that are typically cylindrical or oval shaped. Note that nuclear (N) and subsarcolemmal (S) located mitochondria vary greatly in size and shape. **B** enlargement of intermyofibrillar mitochondria with soft membrane (top pointer) indicative of fusion or fission. Note, the lower pointer indicates clearly defined membranes. **C** illustrates the ordered arrangement of mitochondria in longitudinal sections on the left and irregularly shaped mitochondria can be observed in obliquely sectioned samples on the right. Scale bars, 5 μm (**A**); 0.5 μm (**B**); 2 μm (**C**). (Fig. 4: Journal of Molecular and Cellular Cardiology by Elsevier [25]. Reproduced and lettering modified with permission of Elsevier in the format Journal/Magazine via Copyright Clearance Center)

Cardiac mitochondria typically range between 0.5 and 1 µm in width and 1–2 µm in length [25, 46, 62]. For reference, ventricular cardiomyocytes are large rod-shaped cells with dimensions of 120 × 20 × 10 μm. In the heart, mitochondrial morphology is closely coupled to their localization within subsarcolemmal, perinuclear, or intermyofibrillar space, which have distinctive oxidative demands (Fig. 1A). Hom and Sheu clearly illustrated the morphological variability mitochondria can adopt in adult rat ventricular cardiomyocytes by the ongoing process of fusion and fission (Fig. 1B) [25]. Interfibrillar mitochondria are typically ovular or cylindrically shaped, orientated in longitudinal rows between the myofibrils that are roughly aligned to each sarcomere (Fig. 1C, left panels). The proximity between mitochondria and contractile machinery allows the efficient delivery of ATP to the sites where energy demands are high. Subsarcolemmal and nuclear mitochondria are more varied in size and shaped akin to rods, globules, or horseshoes (Fig. 1C, right panels).

Since mitochondria cannot be synthesized de novo, they are instead derived from existing organelles through the careful balance of fusion and fission, and require the import of proteins and lipids [57]. Fission and fusion have been described elsewhere in detail [25, 36, 75]. Briefly, fusion of the outer and inner membranes of adjacent organelles enables mitochondrial matrix contents to mix, increasing maternally inherited mitochondrial DNA (mtDNA) and promoting the transmission of membrane potential and calcium signals across the length of a cell [25, 75]. In contrast, fission fragments a mitochondrion into daughter mitochondria, and the smaller size enables their efficient trafficking to parts of the cell where energy requirements are high [25, 36]. This is a controversial point given the spatially restricted nature of cardiomyocyte architecture. Tipping the balance towards an increase in fission or decrease in fusion disrupts the highly controlled process and gives rise to fragmented mitochondria, or the reverse result in the appearance of elongated interconnected mitochondria [25]. The latter morphology, as detailed by Wakabayashi [75], forms in instances when ATP demand is lowered which reduces the number of mitochondria per cell and their total surface dimensions. Although it is unclear whether this applies solely to the ‘uniquely’ highly ordered mitochondria in cardiac cells or to the more dispersed cytoplasmic distribution of mitochondria in other cell types such as liver parenchymal cell [60]. Fission and fusion are essential processes to maintain energy production and alleviate oxidative stress (in the case of fusion), whilst also ensuring cell growth and development (in the case of fission). Therefore, if the ratio of fusion and fission are compromised, this can lead to cardiac dysfunction and disease onset, in particular, heart failure [20].

In the current body of literature, an increase of mitochondrial size can be attributed to mitochondrial swelling, fusion or impaired autophagic events, although their exact contributions to the formation of ‘enlarged mitochondria’ which are also commonly referred to as ‘giant mitochondria’ or ‘megamitochondria’ remains controversial. Giant mitochondria and their association with heart biology and pathobiology forms the focus of this review. We also describe instances of giant mitochondrial inclusions from skeletal muscle cells which serve similar contractile functions to the heart and provide an enlightening viewpoint not previously demonstrated in heart muscle. It should also be noted that terminology relating to giant mitochondria has been historically inconsistent with megamitochondria, large or enlarged mitochondria and special mitochondria also mentioned in reference to similar structures in the literature. Herein, we review the available literature on this mitochondrial size aberration including what one needs to understand regarding how giant mitochondria are defined. We provide structural evidence that leads to use of this terminology. Briefly, giant mitochondria are morphological adaptations that on average extend the size and shape by factor of 3–4 compared to normal mitochondria in muscle cells. As such, this thematic paper aims to summarize the relevant literature on the occurrence of giant mitochondria in cardiomyocytes and demonstrate their importance in research and clinical relevance. A summary of the experimental models where giant mitochondria was reported is provided in Table 1.

**Table 1:** Giant mitochondria dimensions in cardiomyocytes

Author (Refs.)	Species	Experimental model	Anatomical location	GM Length (L) × width (W)
Kraus and Cain [33]	Human	Primary cardiomyopathy with no history of alcohol abuse	Right ventricle septum	> 30.0 µm length (surface area 44–72 µm^2^)
Arbustini et al*.* [4]	Human	Dilated cardiomyopathy with mtDNA mutation	Right ventricle	> 6 sarcomeres in length*
Kanzaki et al*.* [31]	Human	Dilated phase of hypertrophic cardiomyopathy	Left ventricle	13.0 × 5.0 µm*
Tandler et al*.* [68]	Human	Restrictive cardiomyopathy	–	~ 14 × 3 µm
Slautterback [62]	Avian	Canaries, Sparrows, Zebra Finches, Quail and Geese	Ventricle	2.5 × 4.0 µm 0.5 × 9.0 µm
Wollenberger & Schulze [78]	Dog	Young adult shepherd dogs with aortic banding induced chronic heart failure	Left & right ventricle	> 10.0 µm length
Liang et al*.* [38]	Mouse	C57BL/6 mice at 4- and 24 month-old	Ventricle	4.0 × 0.5 µm*
Song et al*.* [63]	Mouse	Drp1 null hearts	Left ventricle	3.5 × 1.5 µm*
Woodall et al. [79]	Mouse	12 month-old mice carrying proofreading defective mtDNA polymerase γ (POLG) models, and POLG crossed with human parkin over-expression (POLGxParkin-Tg)	Whole heart	1.5 × 2.0 µm*
Coleman et al*.* [14]	Mouse	C57BL/6 female mice at 6 month, 17 month and 27 month-old were exposed to short- (6 weeks) or long-term (10 month) running schedules	Left ventricle	27 month-old: 4.0 × 5.0 µm*
Mikami et al. [45]	Mouse	Isolated neonatal ventricular myocardial cells	Ventricles	5–10 times normal size
Alexander et al*.* [3]	Mouse	C57Bl mice fed different liquid diets mimicking alcoholic cardiomyopathy	Left & right ventricle	11.0–13.0 µm length
Tandler et al*.* [69]	Mouse	C57Bl/6 adult males with systemic deletion of Klf15 (kruppel-like factor 15)	Left ventricle	Up to 14 µm length
Laguens & Gomez-Dumm [34]	Rat	Adult male Wistar rats were exposed to 60-, 90- or 120 min swimming durations	Left & right ventricle	90 min: 5.5 × 1.0 µm* 120 min: 8.0 × 1.2 µm*
Eppenberger-Eberhardt et al*.* [16]	Rat	Isolated and cultured adult ventricular cardiomyocytes from 2month-old rats Para-crystalline inclusions observed	Ventricle	2.5 × 1.0 µm*
Stenger & Spiro [65]	Rat	Young animals. No specific age, strain or other conditions were detailed	Papillary muscle	5.0 × 1.0 µm*
Sun et al*.* [67]	Rat	Hypoxic perfusion	Right ventricle	12.0 × 3.0 µm*
Bakeeva et al*.* [5]	Naked mole rat	Naked mole rats aged 6 months, 3 years, 5 years and 11 years. Para-crystalline inclusions observed	Left ventricle	5.y.o: 3.2 × 3.1 µm* 11.y.o: 2.2 × 2.5 µm*

Brief summary of the reported mitochondria size (or changes) in different experimental or clinical conditions. Note, not all articles reviewed in this article report on quantitative mitochondrial width and length changes. In some of the reviewed work, the authors attempted to extrapolate the size of the mitochondria if sufficient information was provided as a reference point. A standard reference point, where a scale bar was not provided, is the sarcomere which is estimated to be ~ 2 µm. Extrapolated dimensions are indicated with an asterisk (*). For comparative reasons, the size of normal mitochondria in cardiomyocytes ranges between 1–2 µm in length and 0.5–1 µm in width

## Giant mitochondria in the normal heart

**Fig. 2:**
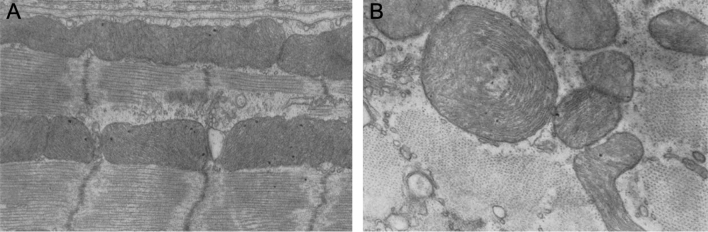
Transmission electron microscopy micrographs of enlarged mitochondria in rat papillary muscle derived from the left ventricular myocardium. **A** Longitudinally orientated muscle section shows elongated subsarcolemmal mitochondria and **B** transverse section showing concentric arrangement of cristae. End magnifications, × 43,000 (**A**); × 53,000 (**B**). (Figs. 1 and 24: The Journal of Biophysical and Biochemical Cytology by Rockefeller Institute Press [65]. Reproduced and lettering modified with permission of Rockefeller Institute Press in the format Journal/Magazine via Copyright Clearance Center)

To the best of our knowledge, giant mitochondria in the mammalian heart was first shown in 1961 as an incidental finding by transmission electron microscopy investigation of papillary muscles of young rats and dogs [65]. This study aimed to describe the detailed ultrastructure of the mammalian cardiac muscle with a primary focus on sarcomere organization. The presence of ‘large’ mitochondria was noted but not quantified (Fig. 2). From the images published, we were able to identify a large subsarcolemmal mitochondrion from rat papillary muscle, with an estimated length spanning two and a half longitudinally orientated sarcomeres before being cut off by the image boundary. Estimates of average sarcomere length is 2 µm suggesting the dimensions of this large mitochondrion to be approximately 5 µm in length and 1 µm in width (Fig. 2A) with the presence of shelf-like cristae structures. In a transverse section of the rat heart muscle, large concentrically arranged cristae were also presented, although the authors noted this as a rare occurrence (Fig. 2B).

**Fig. 3:**
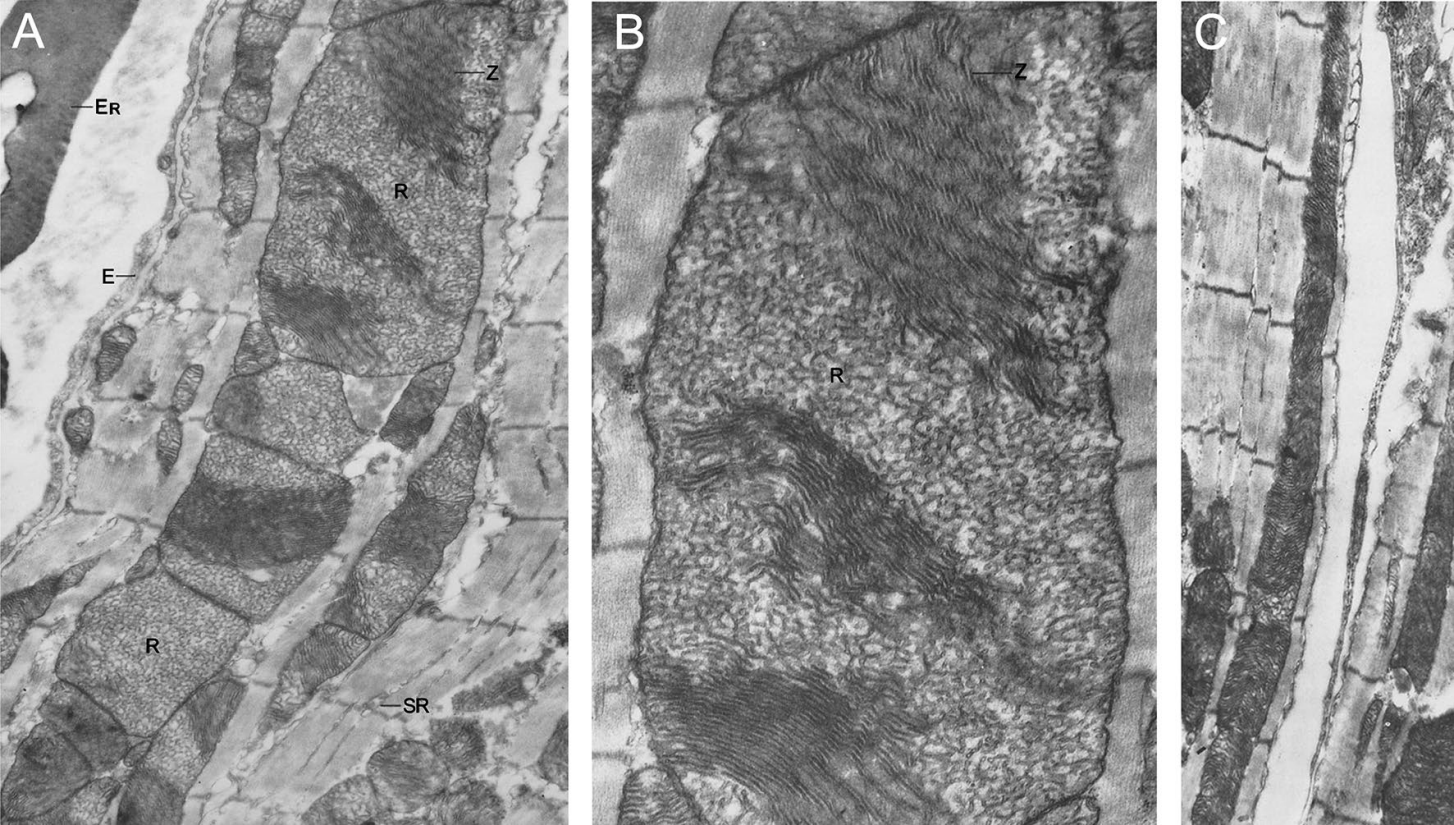
Giant mitochondria in canary myocardial tissue. **A** The size and shape of giant mitochondria varies significantly from **B** block-like to **C** rod-like formation. Giant mitochondria contain both retiform (R) and zig-zag (Z) arrangements of cristae. End magnifications, × 27,000 (**A**); × 60,000 (**B**); × 23,000 (**C**). (Figs. 1, 2 and 14: Journal of Cell Biology by Rockefeller Institute Press [62]. Reproduced and lettering modified with permission of Rockefeller Institute Press in the format Journal/Magazine via Copyright Clearance Center)

Soon thereafter, a specific report on the morphology of mitochondria from the ventricular myocardium of avian models was published [62]. This early study compared the internal structure of myocardial mitochondria in birds with slow 200 beats/min (goose), intermediate 500–600 beats/min (quail) and fast 1000 beats/min (canary, sparrow and zebra finch) heart rates. Of note, the human heart rate is about 60–70 beats/min under basal conditions. In the canary ventricle, a combination of regular and giant mitochondria is present. While the smaller mitochondria (0.2 × 0.5 µm; Fig. 3A) are comprised of predominately shelf-like cristae, the larger and giant mitochondria (2.5 × 4.0 µm, Fig. 3B; 0.5 × 9.0 µm, Fig. 3C) contained both shelf-like zig-zag and retiform (net-like) cristae. Surprisingly, giant mitochondria were not detected in any other avian species irrespective of heart rate even though the authors suggest the possibility of giant mitochondria correlating with the beats per minute. An argument was made that the ‘enormous’ mitochondria, has the advantage of closely packed cristae, carrying greater concentrations of enzymatic units capable of rapidly responding to a sudden increase in energy demand. This idea was seemingly counteracted by an equally likely less efficient system due to diffusion limits of the channels. A rather unsurprising but nevertheless interesting observation in the exemplar electron micrographs presented is the location of the giant mitochondria which appear to mostly localize to the intermyofibrillar space indicative of its role in responding primarily to fluctuations in sarcomeric energy demands (Fig. 3). In addition, the authors also documented the fine zig-zag pattern of the cristae in which small closely packed electron-dense particulates adhere, most likely representing respiratory enzyme complexes.

The importance of cristae organization in cardiac mitochondria became apparent as high-resolution scanning electron microscopy was more readily available. The Hoppel group were one of the first to combine this modality with osmium-mediated cellular extraction to characterize the internal morphology of subsarcolemmal and myofibrillar cardiac mitochondria in normal healthy rats [55]. Riva et al*.* quantified the population of structurally distinct cristae with subsarcolemmal mitochondria possessing predominately lamelliform (plate-like) cristae whereas intermyofibrillar mitochondria were more variable consisting of tubular, lamelliform or a combination of the two cristae structures. The tubular cristae were described as frequently branching and occasionally anastomosing giving rise to a lattice-like structure. These definitions are seemingly comparable to the early description of shelf-like and net-like cristae observed in avian giant mitochondria adjacent to myofibrils [62]. Gross oxidative metabolism measurements from rat hearts showed enhanced oxidative phosphorylation and uncoupled respiration in the intermyofibrillar population [55]. Since oxidative phosphorylation was not the direct result of enzymatic activity in these mitochondria, the authors postulated that the smaller intracristal space within tubular cristae, as compared to the flat conformation, conferred a functional enhancement in proton concentration improving ATP synthase activity which thereby facilitates oxidative phosphorylation. The distinct internal morphology described thus far in normal mitochondria represents an interesting structure–function correlation that warrants further investigation to uncover their biochemical composition. For giant mitochondria, these studies provide a blueprint to begin detailing internal structure, composition and inference of function into its role in the heart.

**Fig. 4:**
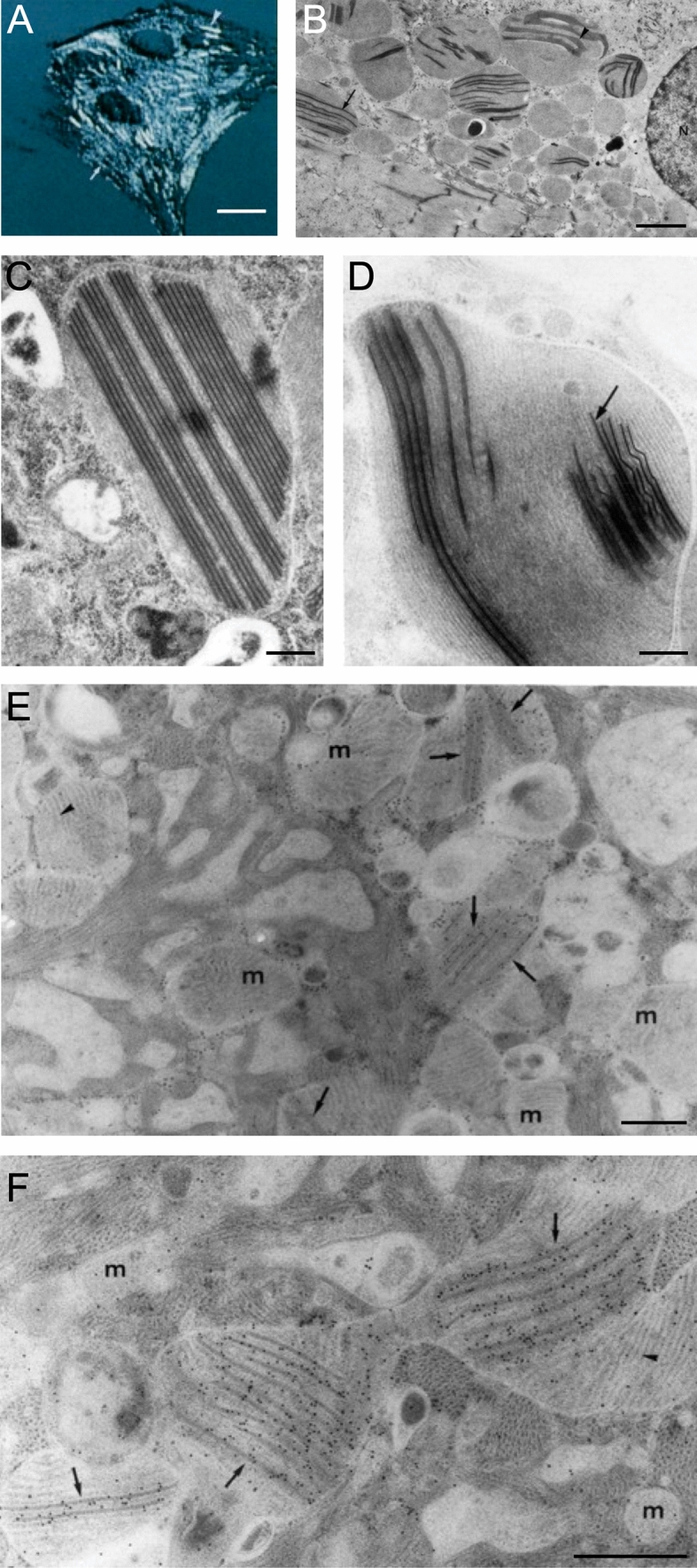
Cultured adult rat ventricular cardiomyocyte (ARVC). **A** Artificially shadowed confocal microscopy image showing giant cylindrical shaped mitochondria (arrowhead). **B** Corresponding low magnification transmission electron microscopy image shows the presence of electron-dense inclusions in giant mitochondria. The inclusions can appear as parallel straight lines (arrow) or sheet liked wavy structures (arrowhead). *N*—nucleus. Periodicity was identified between the parallel straight lines **C** and within the linear structures (**D**, arrow). **E**–**F** ARVCs gold-labelled with mitochondrial creatine kinase antibody are localized at the site of both forms of the mitochondrial inclusions, but largely absent from mitochondria (m) without inclusions. Scale bars, 15 μm (**A**); 3 μm (**B**); 0.45 μm (**C** & **D**); 0.5 μm (**E** & **F**). (Figs. 1F, 3 and 4: Journal of Cell Biology by The Rockefeller University Press [16]. Reproduced and lettering modified with permission of The Rockefeller University Press in the format Journal/Magazine via Copyright Clearance Center)

This poses the question of why are these enlarged or giant mitochondria present in the heart? So far, not much is known as to what triggers the biochemical pathways that give rise to the onset and formation of giant mitochondria in cardiomyocytes. It is possible that giant mitochondria with its increased internal cristae area serves a functional role to adapt to the changing metabolic needs of the heart, such as in physiological conditions described above. The closest study that shed light on this question is by Eppenberger-Eberhardt et al*.* who demonstrated that populations of giant cylindrically shaped mitochondria exhibited strong mitochondrial creatine kinase (mCK) expression in cultured rat adult cardiomyocytes (Fig. 4A) [16]. Transmission electron micrographs of these giant mitochondria showed para-crystalline shaped inclusions located between the cristae membranes (Fig. 4B, C) which were specifically decorated with immunogold labelled anti-mCK (Fig. 4D–F). When the authors added creatine to the culture, the substrate of CK, both giant mitochondria and the para-crystalline inclusions disappeared. These findings suggested that giant mitochondria with the presence of inclusions are perhaps a metabolic adaptation to low intracellular creatine levels, which often occurs under conditions of increased hemodynamic stress and is a characteristic of heart disease (reviewed in [15]).

The low intracellular creatine concentrations and the subsequent low phosphocreatine energy stores are compensated by the accumulation of mCK in the mitochondria. mCK catalyzes the transfer of phosphate from ATP to creatine forming phosphocreatine that can then be transported out of the mitochondria in a process known as the creatine phosphate shuttle. Phosphocreatine serves a functionally critical role as an energy buffer that can replenish the hydrolyzed ATP supply utilized by the contractile mechanism [9]. Thus, the accumulation and storage of mCK in a highly ordered arrangement between the cristae may facilitate the production of phosphocreatine during instances of increased or abnormal energy utilization. Likewise, mCK was confirmed as a major protein present within giant mitochondria bearing para-crystalline inclusions in muscle fibers of patients suffering from a variety of skeletal myopathies [64]. This idea was also underpinned by a more recent study of normal-sized mitochondria derived from the skeletal muscle of patients genotyped with a mitochondrial defect who also presented with para-crystalline inclusions [42, 74]. Amongst the distinct subtypes of para-crystalline inclusions described, the linear type 1 para-crystalline inclusions, identified as stacked sheets forming rectangular inclusions, were visually comparable to those described in the earlier work on adult rat ventricular cardiomyocytes [16]. The study also noted that these inclusions were generally clustered in the subsarcolemmal space and around a nucleus, which serves a functional purpose to allow for de novo synthesis of functional CK protein [74]. Perhaps, the same could be said for the mitochondria in cultured cardiomyocytes which also appear close to the nucleus (Fig. 4B).

## Giant mitochondria in the ageing heart

The ageing heart undergoes a progressive series of physiological changes often associated with a lower tolerance or capacity to adapt to changing workloads which increases the likelihood of disease development. In long-lived post-mitotic cells, such as cardiac cells, the accumulation of lipofuscin deposits and enlarged mitochondrial morphology are prominent histopathological features of ageing which are both correlated with cardiomyocyte senescence and inefficient autophagy [37, 71]. In the heart, cardiomyocytes become senescent with age due to their accumulation of reactive oxygen species (ROS), which induces oxidative damage within the mitochondria—a universal process found in most cell types [7, 10]. Under normal circumstances, damaged mitochondria are recycled by the process of mitophagy [30, 37, 38, 71, 72].

Mitochondria isolated from elderly rat ventricular myocardium were previously shown to exhibit reduced oxidative phosphorylation compared to adult mitochondria [17]. However, this functional impairment was also accompanied by a reduction in the overall yield of mitochondria from aged hearts, and both findings are limited to the interfibrillar mitochondria. No change in yield or function was observed in isolated subsarcolemmal mitochondria. A later study by the same group revealed no differences in cristae morphology between young and ageing cardiac mitochondria [56]. These results suggest that a mechanism independent of the inner membrane architecture may hold the key to oxidative metabolism in the ageing heart. On reflection, the reduction in mitochondrial yield may be attributed to the presence of giant mitochondria which were unlikely represented in this study. Giant mitochondria are fragile, and are often considered to be damaged, organelles that may be lost during the homogenization or protein digestion treatment regime or are co-sedimented along with the nuclei and sarcomeric contents [19, 26, 32]. This still leaves the question open as to why giant mitochondria are present in the ageing heart and whether they remain functional.

**Fig. 5:**
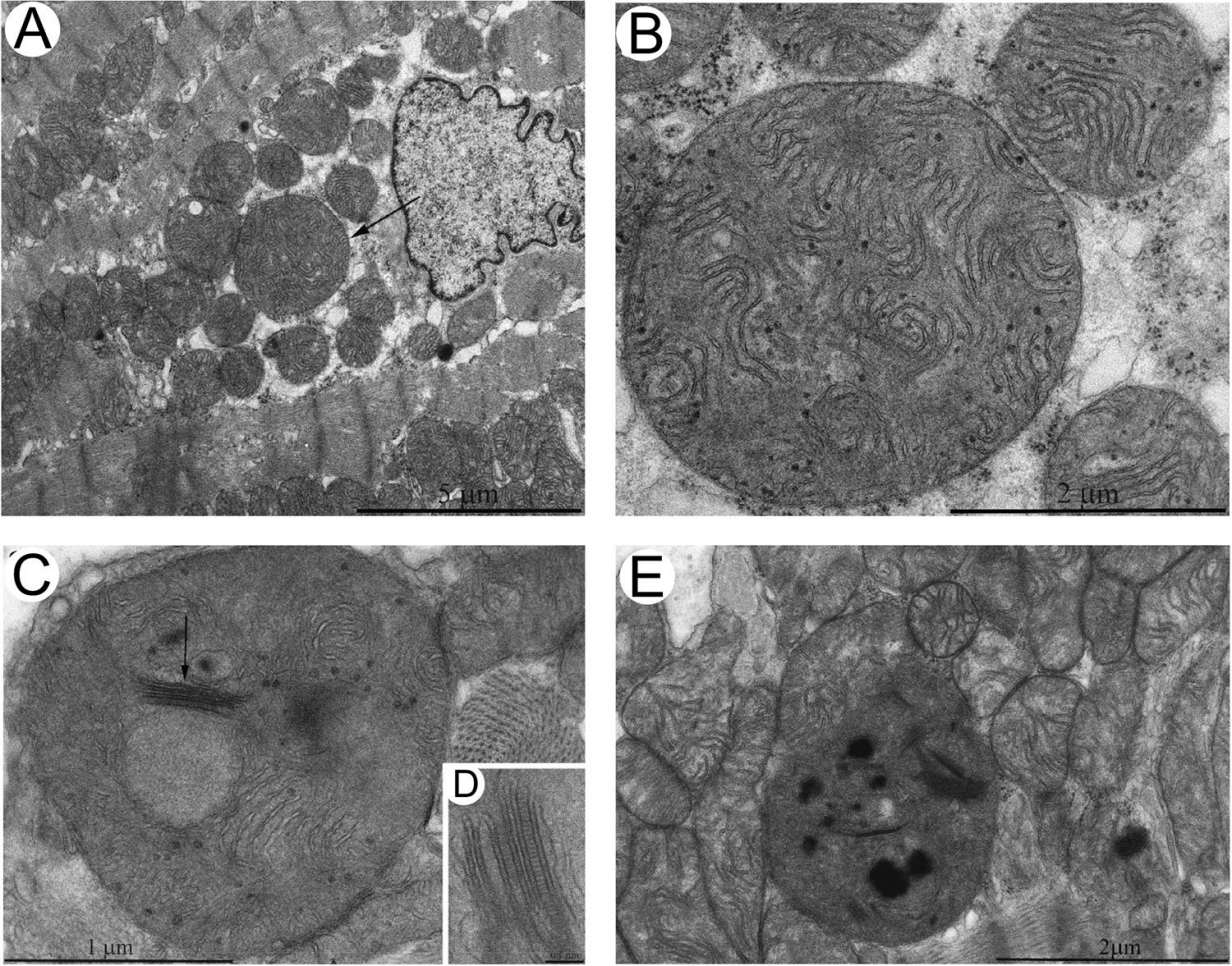
Electron microscopy observations of mitochondria in the naked mole rat. **A** shows the presence of a large mitochondrion (arrow) at 5 years of age. Note, the high number of normal-sized mitochondria surrounding the enlarged mitochondrion. **B** High magnification of the giant mitochondrion from **A** which illustrates packed wave-like cristae. Note the electron-dense granules scattered throughout the mitochondrial matrix. **C** At 11-year-old, the large mitochondria show inclusion of highly ordered cristae bundles and a clear reduction in cristae packing. **D** High magnification of the cristae bundles in **C** which are arranged in parallel, with a track-like appearance between the double-membraned structures termed membrane junctions or intra-crystal junctions. **E** Overview of the disrupted mitochondria ultrastructure in 11 year-old naked mole rats with possible sheet-like inclusions. Scale bars, 5 μm (**A**); 2 μm (**B**); 1 μm (**C**); 0.1 μm (**D**); 2 μm (**E**). (Figs. 5 and 8: International Journal of Molecular Sciences by MDPI [5]. Reproduced and lettering modified with permission of MDPI in the format Journal/Magazine via MDPI Open Access Policy)

The morphology of enlarged mitochondria varies significantly with age and species (Table 1). Bakeeva et al*.* described the ultrastructure of mitochondria in naked mole rats as a model organism for understanding healthy longevity given their long lifespan of more than 30 years and near complete absence of cardiovascular and neurological disorders [5]. Enlarged circular mitochondria, 2–3 times the normal size, and curved ring-like cristae organization was noted as early as 5 years old in the naked mole rat myocardium (Fig. 5A) [5]. Similar ring-like arrangements were previously observed in rat papillary muscles (Fig. 2), although the rat cristae were more concentric and tightly packed than those found in the mole rat (Fig. 5B) [65]. Only as the mole rat reaches maturity at 11 years old, do mitochondrial structural changes begin to appear. These changes are marked by a more electron transparent matrix in normal-sized mitochondria and the loss of cristae in large mitochondria (Fig. 5C and E). These large mitochondria also contain electron-dense para-crystalline structures (Fig. 5D) which are strikingly similar to sheet-like and repetitive parallel inclusions described previously (Fig. 4; [16]) and observed across various pathologies in skeletal muscle [74] and liver [61, 66, 77].

The first mechanistic insight into the formation of giant mitochondria in senescent cardiomyocytes was presented by Terman et al. who postulated that large mitochondria are the product of an inefficient autophagic response in recycling defective organelles [72]. To examine this, autophagy was pharmacologically inhibited with 3-methyladenine in isolated neonatal rat cardiac cells, and was compared to aged cells cultured for 3 months. Aged cells accumulate predominately large mitochondria that retain normal immunoreactivity to cytochrome c oxidase subunit 1 but impaired BrdU incorporation (mtDNA synthesis) and reduced inner membrane potential. This process is accelerated by exposing cells to hyperoxic conditions which induces oxidation-related stress similar to intracellular ROS. In comparison, autophagy-inhibited cells amassed a similar population of large mitochondria but a concurrent increase in small mitochondria which was absent in the aged cells. Remarkably, the population of small mitochondria was reversible and normalized upon withdrawal of 3-methyladenine although the population of large mitochondria remained. This report supports the notion that enlarged mitochondria accumulates with age, and further proposes that these large organelles exhibit defective characteristics that are typically recycled by autophagy but bypasses the process due to its giant size. In a later study, Terman et al. suggested that oxidative damage within large mitochondria may interfere with regular fission, as their size removes them from the autophagic process that can readily and efficiently recycle smaller organelles [71]. The accumulation of large and damaged mitochondria is also likely to continue to produce cardiotoxic ROS, similar to hepatic cells [6, 75].

In a normal physiological system, damaged mitochondria of cardiomyocytes undergo fission where the dysfunctional mitochondrial fragments are eliminated by mitophagy via PINK1 (PTEN-induced putative kinase protein 1), a serine/threonine-protein kinase sensor that resides on the mitochondrial membrane [38]. PINK1 activates parkin which in turn recruits autophagosomes to sequester and deliver the defective mitochondria to the lysosomes for degradation. The accumulation of these receptors promotes the subsequent engulfment of damaged mitochondria by autophagosomes. If the damaged mitochondria fail to be recycled by autophagosomes they can fuse with healthy mitochondria [63], and thus contributing to the formation of giant mitochondria. However, the importance of parkin in this physiological process seems to be disputable [79]. A mouse model of cardiac ageing induced by mtDNA mutation showed cardiac hypertrophy remained unchanged when parkin was globally deleted or over-expressed. In contrast, the original mutant mtDNA mouse model showed enhanced mitochondrial mitophagy in aged hearts which maintained normal mitochondrial respiration. The authors postulated that preserved respiratory function may be partially attributed to the formation of giant mitochondria containing normal mtDNA which acts to dilute the damaged mtDNA—possibly an adaptive cardioprotective function. These giant mitochondria seem to range in size from 2 to 4 µm in length with preserved electron density within the cristae.

Finally, Liang et al. demonstrated that ageing in mouse cardiac cells is associated with reduced autophagosome number, decreased mitochondrial turnover, and formation of giant mitochondria [38]. The overall increase in parkin and ubiquitin expression in aged hearts are attributed to the large number of smaller mitochondria rather than giant mitochondria present in the same sample. The low levels of parkin expression in giant mitochondria are indicative of this organelle bypassing degradation. When combined with a reduction in Drp1 (Dynamin-related protein 1) expression and inactivation of Drp1 by Ser-637 dephosphorylation, mitochondrial morphology is driven towards a fusion state. Conditional knockout of Drp1 in mice suppresses fission, and drives mitochondrial hyperfusion that appears alongside cardiomyocyte necrosis and fibrosis, which are common characteristics of dilated cardiomyopathy [63]. Thus, the presence of giant mitochondria in ageing and disease can be considered an imbalance between mitochondrial fission and fusion towards the latter state, combined with reduced formation of autophagosomes that becomes inefficient in clearing the large organelles. In all, this promotes cardiomyocyte necrosis with resulting fibrotic replacement which are the hallmarks of heart failure.

So far, we have described the formation of giant mitochondria in ageing cardiac models. Navratil et al*.* suggests another possible mechanism using 3-methyladenine to inhibit mitochondrial fusion in skeletal muscle myoblasts [48]. Giant mitochondria were evident as early as 10 days after 3-methyladenine treatment with ovoid or spherical shaped organelles ranging from 1 to 5 µm in size and 3–10 times the thickness of a normal mitochondria. Using fluorescent labels, they were able to clearly identify fusion of normal mitochondria with exchange of matrix content, however, giant mitochondria do not fuse with either normal or other giant organelles. The process of fusion is strongly dependent on the inner membrane potential which decreases with the size of the organelle. Membrane potential in giant mitochondria was impaired by almost five-fold. These findings contrast with the above study [79] but together implies that giant mitochondria are possibly formed in an attempt to dilute mitochondrial damage, but once they become giant mitochondria, they do not further exchange their damaged content with normal organelles and this latter stage impairs their capacity to restore normal function, possibly in addition to inefficient mitophagy.

For completeness, the role of autophagy and the connected process of mitophagy should not be overlooked when attempting to decipher the role of giant mitochondria. Ageing is accompanied by a decline in cellular quality control pathways, mediated by autophagy and mitophagy, that lead to the respective accumulation of dysfunctional cellular components and organelles [39]. These pathways are key contributors in the aging heart where their diminished function is underscored by accelerated ageing including dysfunctional or enlarged mitochondria with dense matrix. The preservation of autophagy, and by proxy mitophagy, has been shown in several pre-clinical models to reduce the ageing phenotype with the potential benefit that protects cardiac function and lifespan [39]. Future high-resolution electron microscopy studies will shed light on whether age-related structural changes resemble the giant mitochondrial architecture observed in heart disease, which we will describe in more detail below.

## Giant mitochondria and physical activity

The ageing heart has thus far provided some mechanistic insights into the formation of giant mitochondria. Further investigations by the addition of chronic stress, in the form of exercise, to older hearts suggest that giant mitochondria ‘appear to have developed as a result of fusion between adjacent hypertrophic mitochondria followed by a sequence of progressive degenerative changes’ [13]. The stress-induced changes were characterized by a reduction in matrix electron density and loss of cristae with dense granular inclusions.

During exercise, myocardial energetic demands increase with a correspondingly rapid change in mitochondrial morphology to maintain cardiac homeostasis. In 1967, Laguens and Gomez-Dumm established that rat cardiac mitochondria begin to transform (i.e., enlarge) after 60 min of constant aerobic swimming exercise [34]. After an additional 60 min of swimming, a further 25% expansion in size coupled with a 4-times increase in the larger mitochondrial population was observed resulting in overall increased mitochondria volume per unit area in the cytoplasm. Enlarged mitochondria identified after 90- and 120 min of exercise span approximately 5–8 µm in length. Furthermore, no ultrastructural modifications in any other components of the cardiac cell were identified suggesting that endurance exercise directly modifies the size of mitochondria.

Importantly, if simple swelling was the expected mechanism for creating giant mitochondria, structural alterations in cristae, matrix and/or outer membrane would be expected. No structural abnormalities of the cristae, membrane or matrix density were noted in longitudinal and transverse sections at the 120 min mark compared to controls. The authors also describe the appearance of invaginations involving the double membrane, lateral buddings and the presence of ribosome like structures were observed earlier in the process at the 60 min mark. This is an important observation which may imply replication and fusion of mitochondria together to form these larger structures with new protein synthesis coming from the ribosomes to maintain structural and matrix integrity. We postulate that an increase in load placed on the heart predominately stimulates an increase in mitochondrial size and mass, although what remains unknown is the process by which these large mitochondria are constructed to cope with changing energy demand, and the type of biochemical or metabolic change they incur.

In 1988, Coleman et al. observed in hearts of sedentary aged 27 month-old mice large mitochondria in the vicinity of nuclei that were associated with lipofuscin deposits and were not otherwise present in young 6-month-old hearts [14]. In young mice, exposure to 30 min of exercise/day for 6 weeks amplified the number of ‘hypertrophied’ mitochondria found within the interfibrillar space of hypertrophied muscle fibers. These enlarged mitochondria are characterized by an increased surface area, electron-dense matrix and closely packed cristae. In aged mice, exposure to 6 weeks (short-term exercise) or 10 months (long-term exercise) of 30 min of exercise/day revealed the presence of giant mitochondria characterized as ‘elongated’ and ‘swollen’ indicating loss of cristae and electron lucent matrix. There also appears to be bundled parallel stacked cristae at the center of the mitochondrion which may be the beginnings of para-crystalline inclusions. Giant mitochondria were noted only in the intermyofibrillar space of degenerating myocytes. The findings from Coleman et al*.* [14] and Laguens and Gomez-Dumm [34] appears to be contradictory in their description of giant mitochondria morphology whereby two main hypotheses arise: (1) short-term exercise induces giant mitochondria that are adaptive rather than pathological and maintains a relatively normal morphology; or (2) long-term exercise and/or ageing (27 months) induces a pathological response beyond physiological compensation (observed at 6 months) resulting in the formation of degenerating giant mitochondria.

In addition to the morphological characterization, the authors also performed quantitative histochemistry to measure succinic dehydrogenase (SDH) activity—a key mitochondrial enzyme for the biogenesis of ATP in myocytes [14]. Young exercising mice showed an increase in SHD activity in concordance with increasing exercise duration, indicating that young hearts can readily respond to changing oxidative demands. In striking contrast, SHD levels in old hearts was reduced irrespective of training regime which is a tell-tale sign that the mitochondria of aged hearts have lost their capacity to adapt to changing physiological workload. Although SDH activity was not directly measured on giant mitochondria, at the very least, it connects the reduced adaptability and plasticity of the adult myocardium with the presence of giant mitochondria. It is interesting to note that studies thus far have all implicated interfibrillar giant mitochondria as the intracellular metabolic or oxidative sensor for the contractile apparatus responsible for matching their ATP consumption.

## Giant mitochondria in heart disease

At the ultrastructural level, abnormal mitochondria in diseased human myocardial tissue mainly present with atypical cristae organization, a significant variation in organelle size and even with mitochondrial inclusions [47]. These structural alterations are typically associated with a reduced capacity to generate ATP (oxidative phosphorylation) and are a major source of ROS production as detailed before (see, giant mitochondria in ageing heart), which can induce cellular damage resulting in cell death and disease progression. These mitochondrial structure–function changes, individually or combined, are implicated in important aspects of cardiovascular disease development beyond ATP synthesis including aberrant cellular calcium homeostasis, vascular smooth muscle pathology, myofibrillar disruption, and altered cell differentiation [10].

### Hypoxia and ischemic injury

Mitochondria in cardiac muscle are highly organized with morphological consistency in size and shape that allows them to be paired to each sarcomere (Fig. 1). Since cardiac mitochondria have proven to be structurally highly dynamic organelles under physiological conditions (Figs. 2, 3, 4 and 5), we describe from this point onwards instances of giant mitochondria under various pathophysiological conditions. Sun et al. [67] induced hypoxia in isolated rat whole hearts which reflects ischemic injury in the acute phase of chronic heart disease when prolonged. In as little as 7 min of exposure to hypoxic conditions, hearts showed marked alterations in the mitochondrial population with evidence of hypertrophy, fusion, degeneration and the formation of elongated giant organelles [67]. These giant structures are accompanied by electron lucent matrices and loss of cristae. Interestingly, narrow channels formed along the border of these newly created giant mitochondria appear to connect adjacent organelles and may serve as early evidence of membrane fusion or pinching. Similar to hypoxic rat hearts, giant mitochondria with morphological features including mitochondrial membrane pinching were also observed in chronic heart disease in dogs induced by aortic banding [78]. Although no giant mitochondria were observed in dogs which developed aortic stenosis without overt heart failure. The appearance of giant mitochondria in acute [67] and chronic [78] form of heart failure but not in milder forms of heart disease poses an interesting question regarding their ultimate function—adaptive prior to onset or compensatory after the fact?

### Alcohol-related heart disease

Giant mitochondria in heart failure gained traction when identified in alcoholic heart disease. Particularly from studies examining alcohol-induced toxicity of cardiomyocytes which is perhaps not all that surprising since giant mitochondria were first described in liver-related pathologies where alcohol is a major contributor [11]. Initial ultrastructural changes in the myocardium from alcoholic cardiomyopathy autopsies was detailed by Hibbs et al*.* [24], which described compact enlarged mitochondria with dense intramitochondrial inclusions. Thereafter, biopsies from 58 patients with chronic alcohol-induced heart failure were conducted [2]. The mitochondria in the diseased myocardium were enlarged and swollen to such an extent that they appeared as ‘ghost mitochondria’ with almost near evacuation of their internal contents. Only sparse cristae and dense round bodies remained in the mitochondria. Whether these dramatic ultrastructural changes were the direct result of alcohol toxicity or general heart failure was difficult to tease apart which led to the development of an experimental mouse model of alcoholic cardiomyopathy by the same group [3].

**Fig. 6:**
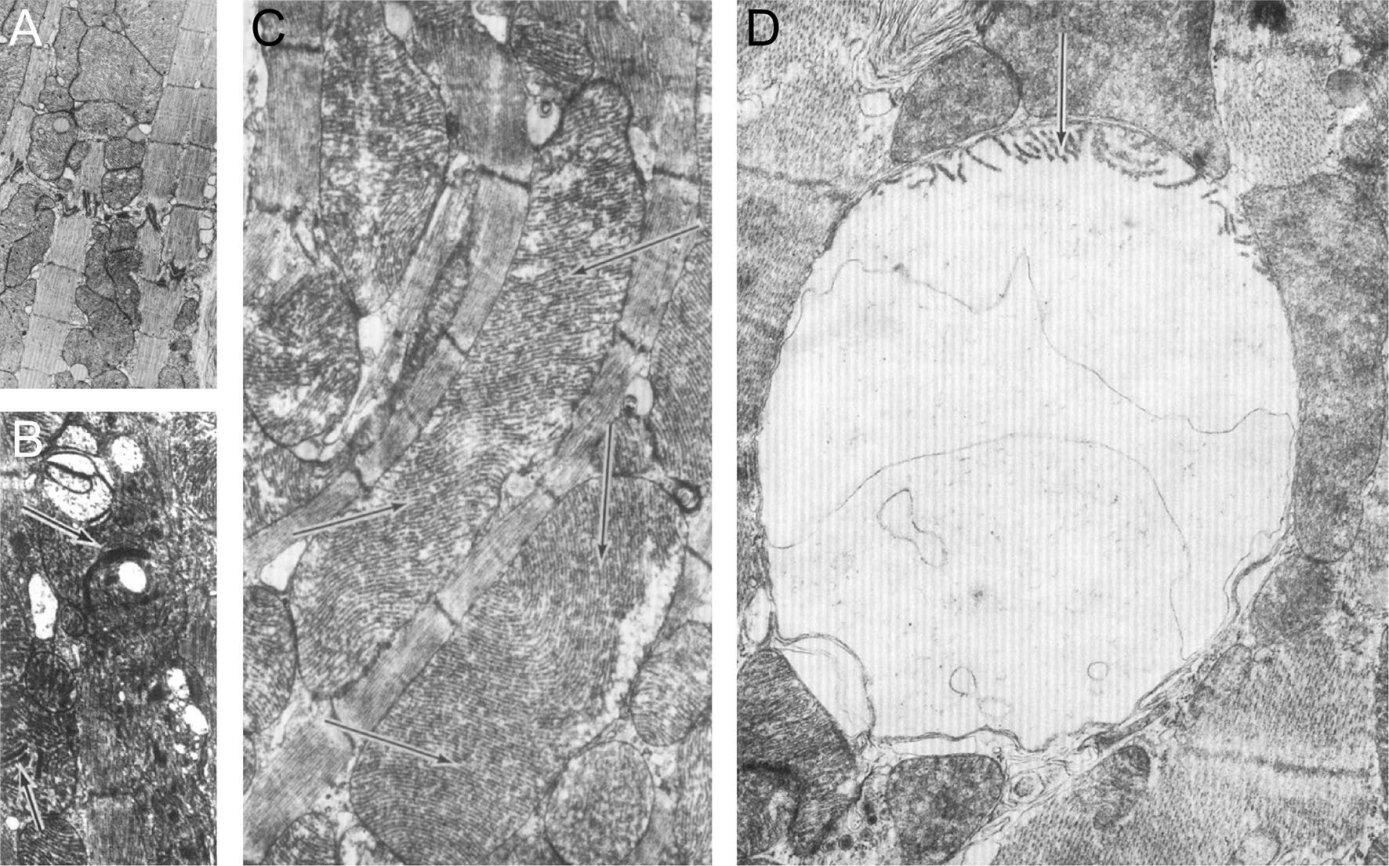
Transmission electron micrographs of giant mitochondria in the ventricular myocardium of alcoholic cardiomyopathy. Mice were fed with an alcohol-heavy diet for **A** 15 or **B**–**D** 25 weeks, consisting of 36% of total calories consumed. **A** Mice fed for 15 weeks with alcohol revealed an increased number and size of mitochondria. The mitochondrial matrix was in general well-preserved. **B**–**D** After 25 weeks of alcohol exposure giant mitochondria, some extending the length of 4–5 sarcomeres, were abundantly present with loss of normal mitochondria structure. In brief: **B** giant mitochondria showing dense cristae (arrow), vacuoles and dense bodies of unknown origin. **C** Depicting two giant mitochondria that possess varying patterns of ‘crystal’ orientation (arrows). **D** In some instances, transformation of giant mitochondria into a huge vacuole was observed. Arrow denotes a few remaining cristae remnants. End magnifications, × 8600 (**A**); × 13,500 (**B**); × 17,500 (**C**); × 25,000 (**D**). (Figs. 2–4: Journal of Molecular and Cellular Cardiology by Elsevier [3]. Reproduced and lettering modified with permission of Elsevier in the format Journal/Magazine via Copyright Clearance Center)

Alcoholic cardiomyopathy in mice was induced by prolonged feeding of a liquid ethanol diet comprised of up to 36% of the total calorie intake [3]. In this study, the authors observed the obvious manifestation of lipid vesicles scattered throughout the cell (Fig. 6A), but perhaps more importantly, the staggering evidence of giant mitochondria that extended across the length of 4–5 sarcomeres (11–13 µm) (Fig. 6C). Single unfused enlarged mitochondria were described by the authors as presenting with ‘a moth-eaten appearance’ which is indicative of cristae fragmentation (Fig. 6A) along with the inclusion of electron-dense bodies and vacuoles (Fig. 6B). Elongated intermyofibrillar giant mitochondria which were clearly formed by hypertrophy and fusion of the outer membranes retained evidenced of the original orientation and arrangement of cristae (Fig. 6C). An important and possibly overlooked subcellular detail of this study is the appearance of giant mitochondria in myocardium exposed to ethanol that comprised 36% of the total calorie intake and not at the lower alcohol levels of 15%. The heart itself does not metabolize alcohol, as such the liver is required to break down ethanol to its metabolic product which are primarily acetaldehydes that can enter the mitochondria where it is oxidized to acetate [76]. Thus, the exposure to circulating acetaldehydes and acetates might be responsible for the formation of giant mitochondria amongst other ultrastructural changes. This process may also be responsible for the increased lipid droplets as acetate enhances the incorporation of fatty acids, normally an important energy substrate, into myocardial lipid deposits.

In an in-vitro model mimicking alcohol-induced cardiomyopathy, Mikami et al*.* examined the effects of acute ethanol treatment on cultured neonatal mouse cardiomyocytes over 24 h [45]. Giant mitochondria, more than five times the size of a normal mitochondria, were obvious at the 1 h mark with evidence of mitochondrial fusion already beginning as early as 5 min of exposure to ethanol. These giant mitochondria were variably shaped and appeared with a fragile outer membrane, an irregularly arranged stacked cristae and a matrix that became electron lucent. Clear visual evidence of mitochondrial fusion of the outer membranes are present. The findings of this study are very much consistent with earlier studies from the Alexander group [2, 3], in which giant mitochondria formed when exposed to 200 mM and 500 mM ethanol but not at 12.5 and 50 mM of ethanol. We provide further interpretation of the process of giant mitochondria formation: (1) immediate aggregation of regular mitochondria occurs upon exposure to ethanol; (2) mitochondria begin to fuse as early as five minutes; (3) highly irregular shaped but unmistakably giant mitochondria populate the cell as early as 30 min; and finally (4) this process was accelerated at high ethanol concentrations that may be associated with more complete diffusion related to alcohol cardiotoxicity. Furthermore, under all ethanol concentrations, glycogen granules gradually increased within the cell cytoplasm up to 30 min of exposure, before a gradual return to normal levels after 24 h of exposure. Since glycogen levels correspond to the formation of giant mitochondria, it may represent an increased energetic need beyond that provided by oxidative phosphorylation or an aberrant utilization of normal energetic pathways, which are then supplemented by the glycogen stores [54].

### Primary cardiomyopathies

The formation of giant mitochondria is not limited to alcohol related heart conditions. One of the largest identified giant mitochondria in the human heart was provided by Kraus & Cain [33]. A biopsy was derived from a 58-year-old man with primary cardiomyopathy and no history of alcohol abuse. Here, intermyofibrillar giant mitochondria frequently extend across 10 and 20 sarcomeres with a length profile of up to 30 µm, and surface area ranging between 44.2 to 72.1 µm^2^. Akin to the internal structures observed in alcohol cardiomyopathy, these giant mitochondria were also rich in cristae arranged in various orientations with diminished matrix similar to the previous description of a fragmented appearance. Intriguingly, these mitochondria are highly variable in shape with an elongated ‘cigar-like’ or irregular branched appearance. Branched giant mitochondria were characterized by a whorl-type arrangement of the cristae while the more common reticular appearance was present in rod-like giant mitochondria. Some giant mitochondria also present with inclusion of granules which the authors describe as glycogen inclusions by a phagocytotic-like process during mitochondrial fusion. Finally, large membrane bound vacuoles, similar to the ‘ghost mitochondria’ in alcoholic cardiomyopathy (Fig. 6), were also present in primary human cardiomyopathy. It was thought that these are the remnants of giant mitochondria degraded by autophagy.

**Fig. 7:**
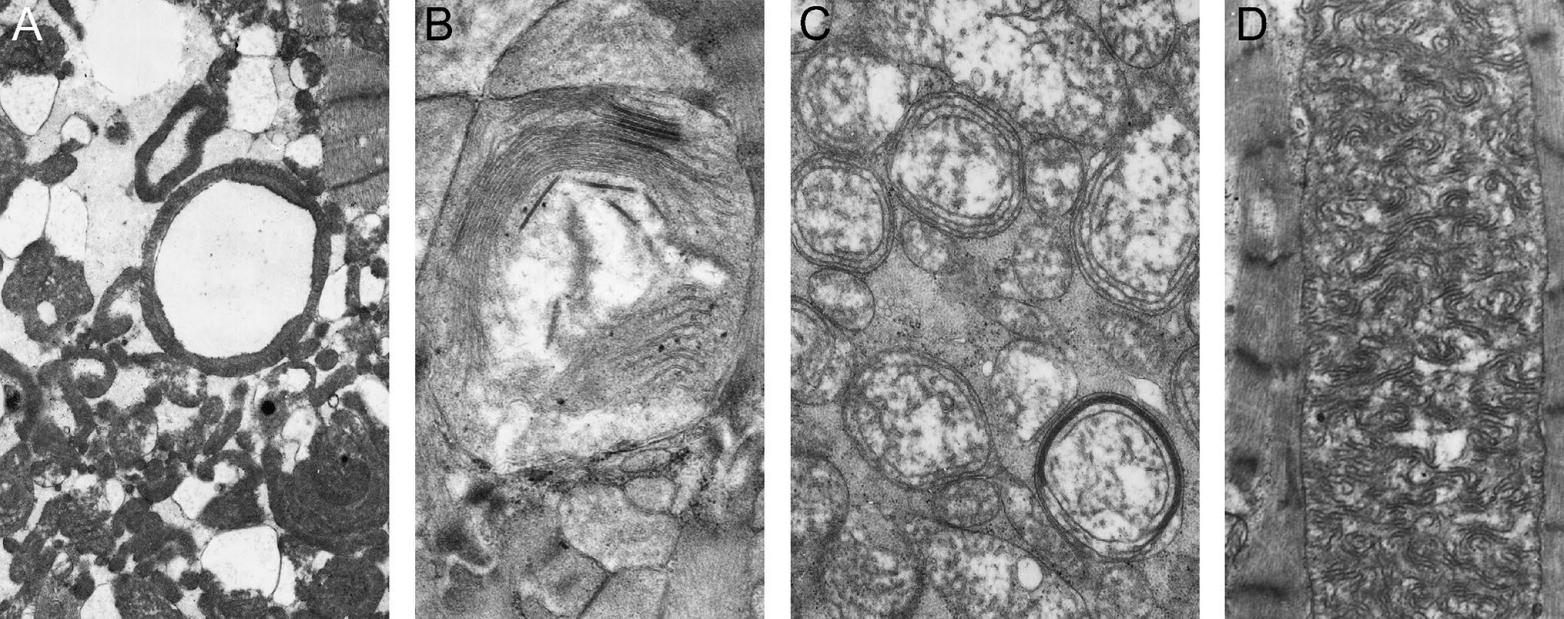
Electron microscopy micrographs showing ultrastructural changes of mitochondria in patients with mtDNA mutations. **A** Ring-shaped mitochondria. **B** Giant mitochondria with membrane fusion and circular cristae. **C** Concentric cristae. **D** Giant ‘organelles’ containing irregularly whorled and undulated cristae. End magnifications, × 5600 (**A**); × 16,000 (**B**); × 9600 (**C**); × 9100 (**D**). (Fig. 1: American Journal of Pathology by Elsevier [4]. Reproduced and lettering modified with permission of Elsevier’s Open Access Content License policy for the American Society for Investigative Pathology in the format Journal/Magazine and subject to proper acknowledgement of the original source)

A similar report, screening 601 dilated cardiomyopathy patients, identified 85 patients who presented with giant mitochondria and crystalloid or osmiophilic inclusion bodies. Of the 85 patients, 19 patients carried mitochondrial (mtDNA) mutations that became the focus of the study with 9 in transfer RNA, 5 in ribosomal RNA, 3 in NADH dehydrogenase (subunit 1) and 2 in NADH dehydrogenase (subunit 2) [4]. The giant mitochondria from these patients were extremely heterogenous in appearance with some being ring-, rod- or ovular-shaped with electron lucent matrix and variable concentric, stacked, or whorl-type cristae arrangements (Fig. 7). The mtDNA mutant patients exhibited reduced cytochrome c oxidase enzyme and NADH dehydrogenase activities compared to both donors and non-mutant dilated cardiomyopathy patients suggestive of perturbations to the electron transport chain. Whether these oxidative capacities can be directly attributed to formation of giant mitochondria, or a downstream effect of mitochondrial mutation is unclear.

Despite the differences in mitochondrial morphology, the occurrence of giant mitochondria in both exposure to alcohol and from mtDNA mutation suggests that there may be some common mechanism of disease that remains to be elucidated. Noteworthy, rat hearts chronically exposed to alcohol consumption demonstrated a significant reduction in cytochrome oxidase activity and cytochrome c oxidase subunit 1 expression [35] which was also found in patients with mtDNA mutations [4]. Chronic alcohol also increased mtDNA damage that may be associated with altered mitochondrial topoisomerase-dependent DNA cleavage and impaired DNA relaxation which prevents mtDNA repair [35]. Although the investigators did not specifically identify mitochondrial structural morphology, it provides some interesting insights by proxy into giant mitochondria biology and suggests that their presence in the above conditions could be the result of damaged mtDNA or an aberrant process in repairing mtDNA.

What is clear is that mitochondrial mutations are not one size fits all as demonstrated by Arbustini et al. [4] and further reinforced by Kanzaki et al. [31] and Houston et al. [27] who also described variable shaped giant mitochondria in other distinct forms of heart diseases. Kanzaki et al. observed giant mitochondria in a patient diagnosed with a dilated phase of hypertrophic cardiomyopathy. The patient displayed concentric circular whorl-like cristae surrounding a central tubular component along with evidence of lamellar undulating cristae. There was also evidence of mitochondrial fusion between adjacent membranes [31]. Houston et al. identified giant mitochondria in a patient with heart failure with preserved ejection fraction. The patient was treated with a cocktail of common heart failure medications that was toxic to the mitochondria (e.g., statins, metformin, aspirin, beta-blockers, glucocorticoids, etc.) until the eventual identification of a mitochondrial missense mutation [27]. The giant mitochondria in this biopsy sample revealed an interesting concentric ring-shaped morphology resembling Fig. 7A. The switch from mitochondrial toxic medications towards antioxidant medications resulted in marked symptomatic improvement. Unfortunately, further investigation after the switching of medication was not conducted to determine whether giant mitochondrial morphology was also reversed.

Giant mitochondria were also identified in cardiomyocytes of Klf15 (kruppel-like factor 15) knockout mice [69]. Klf15 is a negative regulator of cardiac hypertrophy and promotes cardiac lipid metabolism [18], and its deletion seems to interfere with nuclear control of mitochondrial fission but fusion was unaffected [69]. Klf15 knockout produced hypertrophied cardiomyocytes that may in part be due to mitochondrial expansion. The giant mitochondria were found sporadically in the subsarcolemmal space but were much more common in the intermyofibrillar space. Each giant mitochondrion typically span tens of sarcomeres (~ 14 µm length) and are fragmented but have near parallel organization of cristate. What is interesting in this model of giant mitochondria are the evidence of pinching which seems to be a feature of intermyofibrillar mitochondria and was previously identified in hypoxic models [67] but not in human cardiomyopathies [68] where the cristae arrangement are highly compact.

The mechanism of giant mitochondria formation, at least in the Klf15 knockout model, can be attributed to impaired or incomplete fission by membrane pinching which pushes the balance towards promoting fusion. Pinching was later described in detail by Tandler et al. as one of two main fission modalities that effectively prunes the mitochondria into smaller compartments by shrinking the outer membrane which then topologically restricts the inner membrane [70]. Thus, membrane pinching may be a mechanistic attempt to restore the fusion-fission balance presumably prohibited due to its large size and distance around which the fission proteins, dynamin and dynamin-related protein, are required to constrict and contract the mitochondrial membrane [70, 81].

Taking all the above together, giant mitochondria seem to be a common feature in many forms of cardiomyopathy. It is possible that imbalances in fission and fusion ratios, a feature that may start to develop in normal cardiac ageing too, contributes to the common pathways of heart failure.

## Giant mitochondria and physiological adaptation

For completeness, we describe one final intriguing observation of enlarged mitochondria which forms in the muscles of ground squirrels hibernating at 3–5 °C compared to the control group which did not undergo hibernation [46]. Remarkably, enlarged mitochondria were not found in the heart or diaphragm, rather they appear in the skeletal muscle from the forelimb and hindlimbs. The cristae of these mitochondria were more densely packed in the hibernating cohort than that of the controls. In stark contrast, giant mitochondria were found in the hearts of chemically induced hyperthyroidism where metabolic rates are thought to be on the opposite spectrum of hibernation [12]. One hypothesis for the presence of enlarged mitochondria in hibernating skeletal muscle is the maintenance of basal metabolism required to keep the animals alive. Wakabayshi regarded this scenario as an adaptive process to unfavorable environments at the level of the subcellular organelle [75]. One can assume that similar physiological or pathological circumstances may occur such in the intact exercising organ or in muscle injuries of athletes where changes to metabolism and temperature are evident.

## General considerations on cardiac mitochondrial giantism

### Defining the giant mitochondria and limitations in models to date

It is clear that giant mitochondria exist in the heart but the various structure–function aspects that define this organelle remain to be discovered. As Wakabayashi clearly explained, mitochondria should be considered ‘giant’ when its size exceeds that of simple swelling [75]. In a simple experiment, mitochondria isolated from beef heart and rat liver were exposed to hypotonic solution to induce swelling. Once mitochondria swell to 2–3 times its normal size, they lose matrix proteins due to disruption of the outer mitochondrial membrane. As a result, a mitochondrion should be considered ‘giant’ or ‘mega’ once it exceeds the size expected from swelling, all the while, maintaining its membrane integrity.

We have endeavored to collate the literature of evidence that giant mitochondria are found in the heart under a litany of conditions (Table 1). Table 1 summaries experimental conditions that simulate ageing, exercise or disease phenotypes. This review also identified genetic models where giant mitochondria are reported include POLG defect mimicking mtDNA damage [79], DRP1 knockout [63] and Klf15 knockout [69]. Upon closer inspection of the dimensions, only the Klf15 knockout produced the most extreme changes in morphology that most closely resembles our definition of naturally occurring giant mitochondria observed in human pathologies (Table 1). This emphasizes the translational challenges when interpreting cell culture data and whether they respond similarly to the intact organ function and highlights the barrier to study the structure–function of this phenomenon. While in-vitro cultured cardiomyocytes are generally considered suboptimal models of chronic conditions like cardiovascular diseases and hold true when subject to acute high dose toxic stimuli, it remains the most viable alternative that can shed light on the mechanistic underpinnings of giant mitochondria formation. We have described an elegant example in the case of alcoholic cardiomyopathy where in-vitro data provided convincing evidence of the in-vivo conditions further supporting the elaborative experimentation in whole organ and/or humans. Below, we will discuss the importance and implications of variable aspects of giant mitochondrial morphology.

### Fusion and exchange of mitochondrial content

**Fig. 8:**
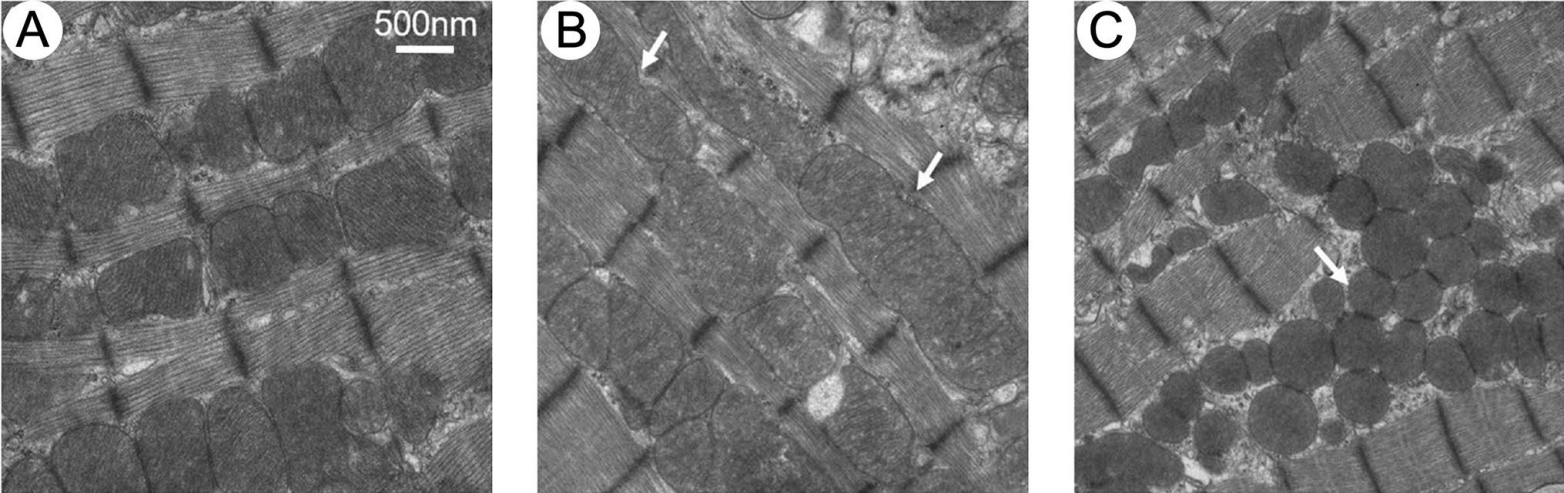
Fusion and fission events of cultured adult cardiomyocytes. **A** normal mitochondria. **B** Fusion of 2–3 mitochondria to form giant mitochondria mediated by Drp1 inhibition. Arrow denotes the area were mitochondria fused. **C** Mfn1/2 (mitofusins 1 and 2) knockout causes mitochondrial fragmentation (arrow). Note that the mitochondria are rounder and their size decreased. Scale bars, 0.5 μm. (Fig. 3: Frontiers in Cell and Developmental Biology by Frontiers Media SA [36]. Reproduced and lettering modified with permission of Frontiers Media SA in the format Journal/Magazine via Frontiers Copyright Notice Open Policy)

There are several possible proposed mechanisms including hypertrophy of individual mitochondrion, fusion of adjacent mitochondria and the suppression of mitochondrion division [75]. The literature thus far, suggests that a combination of fission–fusion imbalance along with incomplete autophagy are strong contributors to the presence of giant mitochondria in the heart (Fig. 8). Another important consideration is that fission–fusion events are often observed in cellular environments that enable high motility of intracellular components [36]. While this may be a possibility for subsarcolemmal mitochondria, it is obviously not the case for the structurally restricted intermyofibrillar mitochondria, where giant mitochondria are most observed, which are arranged in a near crystalline array. Huang et al. suggested a third hypothesis that may allow mitochondria in adult cardiomyocytes to exchange their contents by “kissing” described as the formation of nano-tunnels between adjacent mitochondria that facilitates fusion events and exchange of matrix content [28, 36]. Although this form of mitochondrial exchange seems to be more prevalent in skeletal muscles with mitochondrial defects [74] which perhaps is expected given the columnar like arrangement of the skeletal mitochondria traversing the z-discs of several adjacent myofibrils [51].

### Disrupting the typical energetic arrangement

A further interesting consideration is the parallel alignment of mitochondria within cardiomyocytes. Mitochondria, particularly those localized to the intermyofibrillar space, are closely packed and highly organized parallel to the myofibril and bounded by the z-discs of sarcomere (Fig. 1). This arrangement enables the efficient uptake of substrates required for ATP synthesis and minimizes the distances over which energy substrates is transported to the myofibrils, reflective of a highly functional and efficient microenvironment. While the size of the mitochondria may be important, two recent studies pointed out that the improper organization of the mitochondria within the cell is an equally important factor in liver and heart pathologies [50, 60]. In hypertrophic cardiomyopathy, disorganized mitochondria secondary to the size, was significantly correlated to uncoupled respiration [50]. Consistently, where cardiac ageing and disease are concerned, the highly ordered parallel arrangement of mitochondria are disrupted by the formation of giant mitochondria from several sarcomeres in width to tens of sarcomeres in length which effectively multiplies the diffusion distance.

### Specialized internal structures: cristae, matrix and granules

Kraus and Cain suggested two basic types of giant mitochondria, those presenting with an increase in cristae and matrix content, and those with an increase in cristae but poor matrix quality [33]. Noteworthy, similar suggestions in liver parenchymal cells have been made by Uchida et al. [73], extending to three types of giant mitochondria in which the third type contains para-crystaline inclusions. While the concordant increase in both matrix and cristae are often observed in liver parenchymal cells, cardiac cells are typically matrix-transparent with variable degrees of cristae organization. They argued that giant mitochondria are formed by the process of fusing normal-sized mitochondria as evidence by the disparate orientation of cristae prior to their organization into uniform stacks. But perhaps more importantly, since giant mitochondria are not observed across every physiological condition, this is not a phenomenon that is the result of simple adaptation or compensation. This statement holds true some forty years later. As an example, giant mitochondria were identified in some cases of hypertrophic cardiomyopathy [31] but a significant reduction from normal mitochondrial size was observed in a recent hypertrophic cardiomyopathy population [50]. We encourage those interested to read the discussion laid out by Kraus and Cain [33].

Historically, distinctive cristae morphology and matrix content adopted by giant mitochondria under a multitude of physiological and pathological conditions can be linked back to metabolic function. The classical definition of cristae morphology-function was reported by Hackenbrock through the study of isolated mouse and rat liver mitochondria by transmission electron microscopy [22, 23] and fluorescence anisotropy [58]. These seminal papers described the transition between two states modulated by ADP concentration: a shift from the condensed state, with wide intracristal spacing and a dense contracted matrix compartment, to an orthodox state with compact cristae and expanded matrix. This switching event from occurs when ADP concentration declines during the conversion from respiratory state III to state IV indicating that the ultrastructural transformations may serve to minimize diffusion bottlenecks in ATP synthesis by condensing its internal architecture [44]. Moreover, the transformations in matrix ultrastructure and matrix protein concentration are directly influenced by the respiratory state of mitochondria [58]. Notably, the observed structural changes are reversible pending on their respiratory cycle, underpinning the unique dynamic nature of mitochondria to transform functionally and structurally between metabolic states. Taking those observations together, mechanochemical ultrastructural transformation serve a basis for energy transfer by modulating metabolite diffusion and, in turn, mitochondrial metabolism.

A later study by Manella suggested that the orthodox and condensed states reflected the appearance of lamellar and tubular cristae morphologies, respectively [44]. These very same morphologies were characterized around the same time in cardiac mitochondria by the Hoppel group as being subcellular location specific [55, 56]. It is possible that the morphologies reflect the basal respiratory or metabolic state in which the subcellular organelles reside, switching between condensed and orthodox as needed may partially explain the mismatch between structure and function in earlier works [55]. Indeed, other groups also observed variable degrees of matrix and cristae density during ageing (Fig. 5) as well as a significant loss of one or both features in pathology (Figs. 6 and 7). While one would expect giant mitochondria to be extreme forms of normal-sized mitochondria and transformed to a greater extent, however, this observation suggests otherwise such that normal-sized mitochondria are indistinguishable from the giant mitochondria in terms of their loss or transformation of internal features. We find this both interesting yet unexpected. Does this mean that the biochemistry of giant mitochondria can be readily reflected by evaluations of regular sized mitochondria?

Dense matrix granules are commonly observed intramitochondrial inclusions first described by Palade in liver tissue [52] but it was not until half a century later that a functional role for these dense granules in cardiomyocytes was postulated by Jacob and colleagues [29]. They hypothesized that dense bodies, comprised of glycoproteins, phospholipids and calcium molecules, regulate oxidative metabolism. When metabolic demand is increased, dense bodies migrate towards the inner membrane, where proteins and lipids are incorporated and calcium is released, to facilitate fusion of the inner and outer membranes forming a ‘contact site’. These contact sites serve as a microenvironment of calcium signaling and calcium mediated activation of dehydrogenases. More recently, the contact sites were thought modulate local mCK and hexokinase concentrations in response to mitochondrial metabolic state, and cellular metabolism [83]. The possible role of matrix granules is intriguing, however, there is an absence of literature-based evidence showing their migration or any indications of its composition in cardiac giant mitochondria to-date.

### Are intact giant mitochondria a reversible phenomenon?

Giant mitochondria also show evidence of reversing their formation after the cessation of the physiological stimuli, for example, during exercise. As such their temporary formation may contribute to their inconsistent identification in the literature. Whether the same applies to the alcoholic heart or early stages of cardiomyopathy remains to be investigated. It was noted, however, in chronic disease, that these giant mitochondria may begin to form vacuoles, show fragmented cristae and the matrix is more electron lucent. These structural alterations represent a form of physical inefficiency in overall organelle homeostasis. Whether incomplete autophagic events leads to this physical characteristic as described by Terman et al*.* [72] or whether the enlargement in itself causes incomplete clearance still remains to be resolved and is an important topic of future investigation.

## Conclusion and future outlooks

Dedicated literature outlining the occurrence of giant mitochondria within the human heart are relatively sparse when compared to the abundant literature detailing their presence in skeletal muscle-related disorders, liver disorders, skin ageing, neurological disease and kidney injury. There is no direct explanation for this other than the observation that retrieving heart tissue biopsies is not part of routine diagnostic practice. Considering the importance of mitochondria to ensure the energy-demanding normal function of the heart, we are surprised that this part of the cellular machinery in cardiomyocytes has been poorly investigated. Especially when considering the vast amount of present research focused on targeted therapeutic approaches to restore overall mitochondria functionality [43, 59, 80]. Besides restoring cell and hence organ function, those approaches are also aligned to combat ageing. We know that ageing is one of the strongest risk factors for heart disease, and the world’s population is ageing rapidly. One of the keys to longevity of the heart could be by interventions at the mitochondrial level restoring its normal structure and hence function [40].

From this literature review, it became clear that various hypotheses have been postulated in the formation of giant mitochondria in cardiomyocytes. Swelling, fusion (as part of their dynamic nature), fission–fusion imbalance, division suppression or even as a result of incomplete autophagy (mitophagy) have been put forward as hypotheses that give rise to abnormally enlarged mitochondria. The potential mechanisms that might give rise to enlarged mitochondria are similar to the ones proposed for other organ models. As such, the ‘universal’ mechanisms that give rise to giant mitochondria formation remains an open question for all organs where they occur. Furthermore, questions posed about the reversibility and dynamic nature of this process form an important basis for therapeutics, considering their presence with increased physical activity or in hibernation.

Small animal experimentation and cell culture models provided extensive contributions to the insights collected. This is not a surprise for obvious reasons. ROS, mCK and mitochondrial DNA damage all seem to have a direct or indirect role in the dawn of cardiomyocyte giant mitochondria. A disturbed ROS pathway and autophagy (mitophagy) seem to be the two main commonalties across the different organs where giant mitochondria are present. Likewise, chronic alcohol consumption and ageing seem to be a common factor in their appearance in the heart, liver and kidney. This underpins once more the importance of healthy living regimes by limiting alcohol consumption and reducing oxidative stress by healthy diets and saying farewell to a sedentary lifestyle [7].

**Fig. 9:**
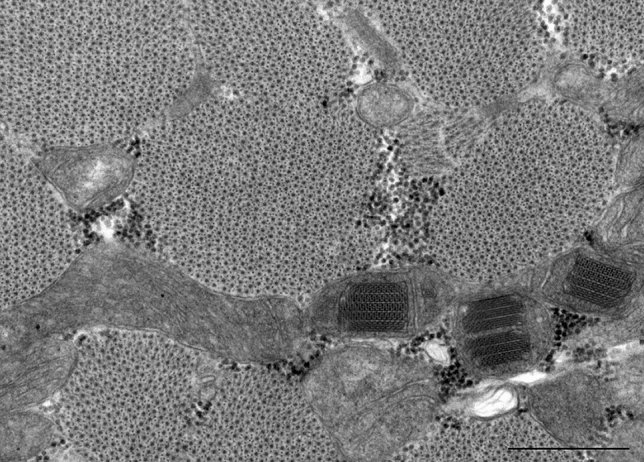
Mitochondria morphology from routine diagnostic imaging. A myopathic biopsy was acquired and sent to clinical pathology services for routine histologic and molecular characterization. High-magnification transmission electron micrograph shows several interesting features including enlarged mitochondria, mitochondria with inclusions, abnormal cristae and matrix interspersed between transverse sectioned highly ordered muscle fibers. Of note, in this example, the patient was diagnosed with focal myonecrosis and underpins the importance of examining muscle tissue for mitochondrial aberrations in diagnostic settings. Scale bars, 0.5 μm—end magnification, × 60,000. (Fig. 1: Australian Microscopy and Microanalysis Newsletter [21]. Reproduced and lettering modified with permission of the copyright holder)

To bridge the knowledge gap of what is known in other organs on giant mitochondria we propose a rigorous approach in which biopsies or explanted tissues are collected and examined across the different heart pathologies. In time, this will enable researchers to build a fuller picture of the omics at the molecular, cellular and tissue level to eludicate mechanism underpinning mitochondrial aberrations, more specifically giant mitochondria. With present methodologies only small quantities of heart tissue is required to perform rigorous screening in order to disclose the biomolecular and cellular make-up of cardiomyocytes. Histological stains such as modified Gomori trichome or Janus green and elaborated with volume electron microscopy similar to the approach to screen for skeletal muscle pathologies [8], would map fine structural organelle changes and aid insights in different cardiomyopathies where giant mitochondria are present. Defining if major components of cristae are altered in giant mitochondria from different pathologies using immunoEM for instance would provide insight into how mechanistic functions may be changed. Figure 9 is an exemplar of the merits of this approach showing the detailed morphology of both enlarged mitochondria and mitochondria with specialized inclusions (i.e., crystal-like) from a myopathic biopsy that is acquired by standard transmission electron microscopy in the diagnostic setting [21]. Current studies on human cardiac tissue by our team are underway inventorying enlarged mitochondria in different heart disease settings.

Structure and function operate hand-in-hand requiring the examination of mitochondrial respiration using high throughput seahorse assays of isolated giant mitochondria and the advent of subcellular spatial omics with associated subcellular imaging technologies provides additional functional physiological insights that can be directly correlated with structural evaluations. Importantly, recent developments in mitochondrial respirometry from frozen tissue has placed us one step closer to being able to measure oxidative function of electron transport chain complexes which restores up to 95% of maximal respiratory capacity often lost during the freeze–thaw cycle [1]. Perhaps one of the more important questions that have not been addressed thus far is whether giant mitochondria utilize different substrates as fuel which is a well-established mechanism in disease [41]. Finally, downstream targeting of fission and fusion proteins in tissue where giant mitochondria are present using immunoEM or subcellular in situ mapping would also provide informative mechanistic insights into the altered fusion-fission theory.
